# Germline variation networks in the PI3K/AKT pathway corresponding to familial high-incidence lung cancer pedigrees

**DOI:** 10.1186/s12885-020-07528-3

**Published:** 2020-12-09

**Authors:** Huan Lin, Gong Zhang, Xu-chao Zhang, Xin-lei Lian, Wen-zhao Zhong, Jian Su, Shi-liang Chen, Yi-long Wu

**Affiliations:** 1 Guangdong Lung Cancer Institute, Guangdong Provincial People’s Hospital and Guangdong Academy of Medical Sciences, 106, Zhongshan Er Rd, Guangzhou, 510080 China; 2 The Second Affiliated Hospital of Guangzhou University of Chinese Medicine, 111, Dade Rd, Guangzhou, 510120 China; 3 MOE Key Laboratory of Tumor Molecular Biology and Key Laboratory of Functional Protein Research of Guangdong Higher Education Institutes, Institute of Life and Health Engineering, Jinan University, 601, Huang-Pu Avenue West, Guangzhou, 510632 China

**Keywords:** PI3K/AKT pathway, Familial lung cancer, Germline variation networks, Whole-exome sequencing

## Abstract

**Background:**

There were scarcely germline variants of familial lung cancer (LC) identified. We conducted an study with whole-exome sequencing of pedigrees with familial lung cancer to analyze the potential genetic susceptibility.

**Methods:**

Probands with the highest hereditary background were identified by our large-scale epidemiological study and five ones were enrolled as a learning set. The germline SNPs (single-nucleotide polymorphisms) of other five similar probands, four healthy individuals in the formerly pedigrees and three patients with sporadic LC were used as a validation set, controlled by three healthy individuals without family history of any cancer. The network of mutated genes was generated using STRING-DB and visualized using Cytoscape.

**Results:**

Specific and shared somatic mutations and germline SNPs were not the shared cause of familial lung cancer. However, individual germline SNPs showed distinct protein-protein interaction network patterns in probands versus healthy individuals and patients with sporadic lung cancer. SNP-containing genes were enriched in the PI3K/AKT pathway. These results were validated in the validation set. Furthermore, patients with familial lung cancer were distinguished by many germline variations in the PI3K/AKT pathway by a simple SVM classification method. It is worth emphasizing that one person with many germline variations in the PI3K/AKT pathway developed lung cancer during follow-up.

**Conclusions:**

The phenomenon that the enrichments of germline SNPs in the PI3K/AKT pathway might be a major predictor of familial susceptibility to lung cancer.

**Supplementary Information:**

The online version contains supplementary material available at 10.1186/s12885-020-07528-3.

## Background

Previous studies have identified very few germline variants associated with familial lung cancer. It’s half a century since familial lung cancer aggregation was observed. An increased familial risk of LC observed in our previous study provided indirect evidence that genetic factors contribute to susceptibility to LC [1]. This echoed an early observation that the LC exhibited familial aggregation.

In LC, no somatic driver mutations have been found for 20% of cases of adenocarcinoma and 60% of cases of squamous carcinoma [2]. One explanation for the mutational heterogeneity observed in cancer is the fact that genes act together in various signaling and regulatory pathways and protein complexes [3]. Accordingly, a pan-cancer network approach that examines combinations of genes may be necessary [4]. Genetic susceptibility to LC may be polygenic and heterogeneous, conferred by relatively common polymorphisms with low penetrance and modest effect sizes [5]. Germline variations may have an important impact on the etiology of complex trait-related pathways, which cannot be explained by common variants. To date, more than 10 genome-wide association studies have examined inherited susceptibility in LC, and relatively few loci have been confirmed [6–11]. Moreover, the results have shown that there are certain differences in inherited susceptibility in LC between the East and West. However, despite these studies, most of the heritability of LC remains unexplained. In one study using next-generation sequencing, disruptive germline mutation genes were identified between familial and sporadic LC [12]. However, the independent statistical analysis of each genomic nucleotide position in GWAS (Genome-wide association studies) makes it difficult to assess the complex interactions among many genes containing these SNPs.

Emerging studies have shown that many inheritable traits and susceptibilities are not caused by single gene mutations, but by accumulation of SNPs of many functional-related genes. In a recent GWAS study of same-sex sexual behavior, the 5 SNPs identified by traditional single-locus statistical criteria explained less than 1% of the heritability, far less than the actual heritability (25% ~ 32%). This demonstrated that the SNPs of many other genes also contribute to the trait, although the contribution of each SNP is minor [13]. Studies on education attainment-associated genes also revealed numerous SNPs in nearly 100 functional-related genes collectively predict the traits [14, 15]. Hence, in this study, we conducted a WES-based epidemiological analysis of pedigrees with the highest genetic susceptibility in lung cancer to analyse the potential genetic background, especially under the hypothesis that multiple SNPs of a group of functional-related genes provide the familial LC susceptibility.

## Methods

### Study design and participants

More than 1300 patients were screened from 2009 to 2010, and 633 pathologically diagnosed LCs were enrolled as probands. The first-degree relatives of both the patients and their spouses were study participants, yielding 565 spouse pedigrees. We collected information on sex, age, lung disease history, race, occupational exposure, living environment, and smoking history for probands and controls (Supplementary Table S1). A detailed description of this study is given in our previous articles [16]. The goal of this study was to characterize the familial genetic susceptibility of LC risk.

### Statistical analyses

We evaluated the risk factors using step-wise logistic regression with the diagnosis of LC as the dependent variable and the following independent variables: age cohort, sex, lung disease history, living environment, occupational exposure, smoking history, and number of affected individuals as first-degree relatives. Univariate and multivariate-adjusted ORs with 95% CIs of LC were calculated using the binary logistic regression model. The estimates were adjusted for sex, age cohort, lung disease history, living environment, and occupational exposure. All of the statistical tests were performed using the SPSS 17.0. Two-sided *P* values of less than 0.05 were considered statistically significant.

### Exome sequencing

Probands having adenocarcinoma and no less than two first-degree relatives with LC were chosen for exome sequencing because of a highest genetic risk in these patients. Healthy controls were selected by matching demographic factors and levels of exposure to kitchen oil, tobacco and living environment variables. Genomic DNA from the blood and from cancer or para-cancer (normal tissues adjacent to cancer) tissues was extracted with a Tiangen Blood/Cell/Tissue genomic DNA extraction kit (Tiangen). A genome sequencing library was constructed using a NEBNext DNA Library Prep Kit for Illumina (New England Biolabs). Exome capture was performed using a SeqCap EZ ExomeV3-Plus kit (Nimblegen). The libraries were sequenced on Illumina HiSeq-2000/2500 sequencers. High-quality reads passing Illumina filter were kept for subsequent bioinformatics analysis.

### Bioinformatics for next-generation sequencing

The clean reads (adapter trimmed) were mapped against the human reference genome GRCh37/hg19 (downloaded from UCSC Genome Browser) using FANSe2 algorithm [17] with the parameters -E4 -I0 -S14 -M1. By piling up the mapped reads, genomic positions with a sequencing depth of greater than or equal to 10× were kept for SNV (single nucleotide variation) detection. SNVs were detected using Fisher exact test against the null hypothesis that the nucleotides at this position were all identical to the reference genome, with a significance threshold of 0.01. This variant calling procedure was experimentally validated for its almost-perfect accuracy and sensitivity [18]. Germline SNPs were defined as nucleotides in para-cancer/blood samples that were different from those in the reference genome. Somatic mutations were defined as SNVs detected in cancer samples but absent in the corresponding para-cancer sample. The workflow is illustrated in the Fig. S2.

Gene annotations were taken from the refflat file downloaded from the UCSC Genome Browser. Nonsynonymous germline SNPs and somatic SNVs were used for network analyses.

### Network analysis

The network of mutated genes was generated using STRING-DB 9.1 [19] and visualized using Cytoscape software v3.0.2 [20]. To ensure high confidence in the analysis, the minimum required interaction scores were set to “high confidence (0.700)”, and only “experiments, databases and gene fusion” were considered as effective evidence for the PPI (protein-protein interaction) sources. The graph properties of the networks were calculated also using Cytoscape software. KEGG pathway enrichment analysis was performed using KEGG online tools (http://www.kegg.jp/). SVM classification details were described in Supplementary Methods.

## Results

### Identification of pedigrees with high risk of familial LC

To reduce possible bias, we adjusted both the case arm and control arm for sex, age, lung disease history, smoking index, living environment, and occupational exposure.

According to the number of affected individuals among the first-degree relatives of the probands and spouses, the pedigrees were divided into three groups: 0, 1, and 2 or more affected individuals (Table 1). As shown in the table, except for one subgroup with a small sample size in the control arm, the remaining groups showed statistically significant differences. Therefore, we found that the subgroup with a family history of at least two first-degree relatives affected by LC was at highest risk.

**Table 1:** Odds ratios for risk of lung cancer among first-degree relatives

Factors	Case/Control	Crude OR (95%CI)	Adjusted OR^**a**^ (95%CI)	***P***-value
Family history of any cancer				
No	432/438	1.00	1.00	
Yes	201/127	1.60(1.24,2.08)	1.71(1.28,2.28)	< 0.001
Family history of lung cancer				
No	560/534	1.00	1.00	
Yes	73/31	2.25(1.45,3.47)	2.20(1.36,3.55)	< 0.001
*N* of pedigrees with				
0	432//438	1.00	1.00	
1	149/111	1.36(1.03,1.80)	1.55(1.14,2.12)	0.002
≥ 2 any cancers	52/16	3.30(1.85,5.86)	2.65(1.42,4.94)	0.001
*N* of pedigrees with				
0	560/534	1.00	1.00	
1	65/30	2.07(1.32, 3.24)	2.11(1.29, 3.44)	0.001
≥ 2 lung cancers	8/1	7.63(0.95, 61.20)	4.49(0.51, 39.27)	0.029

^a^ Adjusted for sex, smoking index, lung disease history, living environment, and occupational exposure

In Table 2, while comparing patients of squamous carcinoma with small cell LC, family history of disease was not significantly different. However, while comparing patients of adenocarcinoma with squamous carcinoma, a family history of disease in first-degree relatives significantly increased the risk of lung adenocarcinoma (OR = 2.74, *P* = 0.018).

**Table 2:** Risk of family history on lung cancer stratified by histologic characteristics

Histologic characteristics	Family history of lung cancer		Adjusted OR^**a**^ (95%CI)
	No N(%)	Yes N(%)	
Squamous carcinoma	111(94.1)	7(5.9)	1.00
Small cell carcinoma	56(94.9)	3(5.1)	0.90 (0.22, 3.63)
Adenocarcinoma	427(85.7)	71(14.3)	2.74 (1.19, 6.31)

^a^ Adjusted for sex, smoking index, lung disease history, living environment, and occupational exposure

Therefore, we identified pedigrees whose probands had adenocarcinoma and had no less than two first-degree relatives with LC as having a highest genetic risk. The affected individuals were biologically related (Supplementary Table S2).

**Fig. 1:**
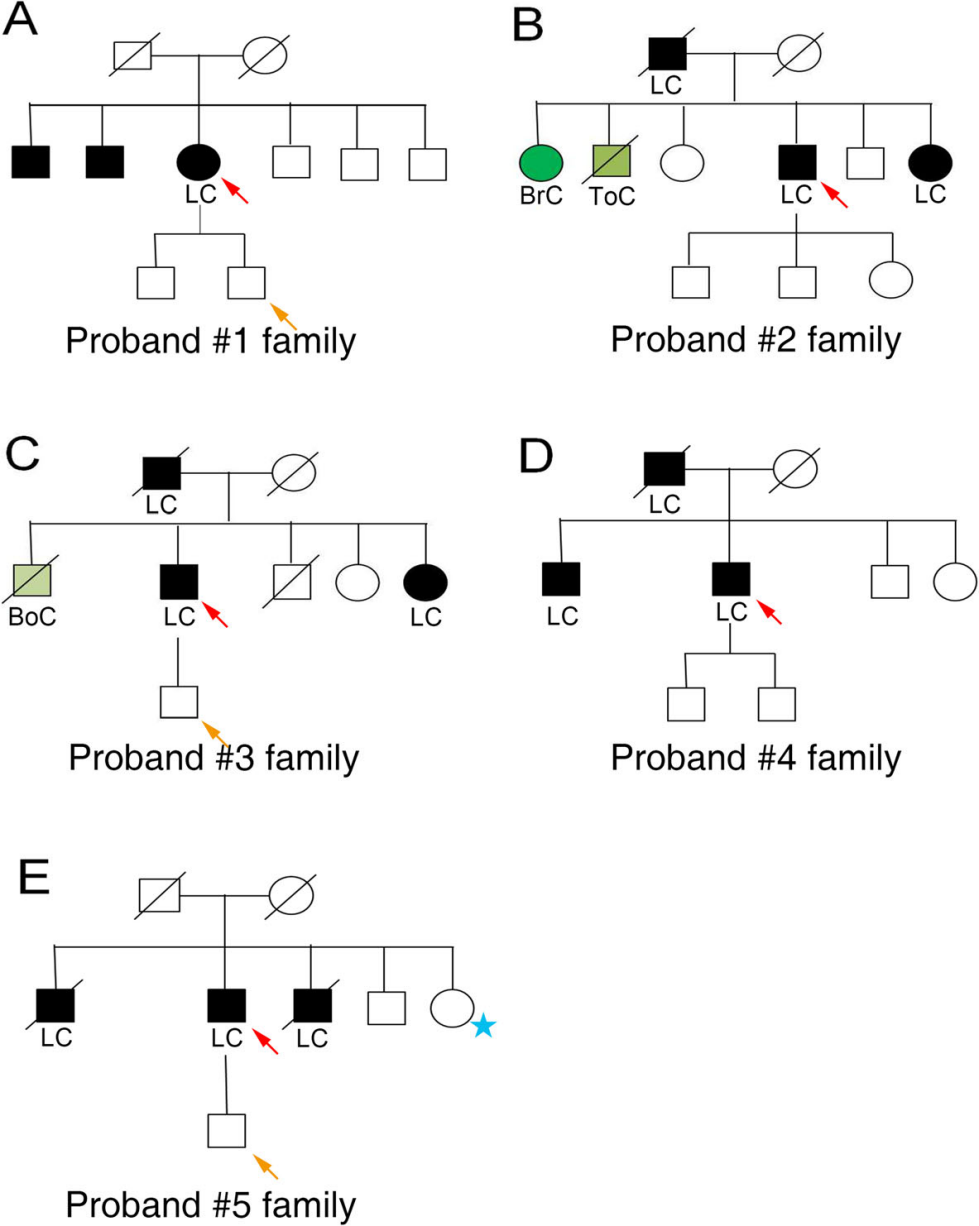
Diseases are given underneath the symbol. Deceased individuals are designated with a slash through the symbol. Cancers in the pedigree are shown (LC = lung cancer; BrC = breast cancer; ToC = tongue cancer; BoC = bone cancer). Red arrows indicate sequenced probands. Orange arrows indicate sequenced healthy individuals in the pedigrees. The cyan star marked the individual who was healthy when enrolled in the study but was diagnosed of LC in the follow-up. See Fig. 2g and Fig. 3

We included five probands as learning sets who were from familial LC pedigrees determined by epidemiological analysis (Fig. 1, red arrows). We also included three healthy individuals without a family history of any cancer as controls.

### Shared somatic mutations and germline SNPs in the probands may not associate with familial lung cancer

We performed WES of both cancer tissues and para-cancer tissues from the five probands. Each sample (cancer and para-cancer) yielded more than 100 million 100-nt reads from the sequencer. 82 ~ 85% of the reads were mapped to the human reference genome, indicating a good quality of the entire sequencing experiment (Supplementary Table S3). The exome capture kit which captures 96 Mb exon and UTR regions; therefore, the nominal average depth of the captured regions reached more than 91x (Supplementary Table S3), providing a good basis for SNV and SNP calling. We identified 727–1033 nonsynonymous somatic mutations (Supplementary Table S4), but none was shared in all five probands, suggesting that shared somatic mutations were not the cause of the familial high incidence of LC. No known driver mutations were found in the five probands, except a KRAS G12V in proband 5. These findings indicated that driver mutations may not explain the high incidence of LC.

We next identified 281 shared germline SNPs among all probands (Supplementary Table S5). However, few PPIs were found among these 281 genes according to STRING-DB; only three subgraphs showed more than five nodes (Fig. S1). No significant enrichment of interactions was found against the genetic background (*P* = 0.102), demonstrating that this network was a random sample from the genetic background. Gene ontology enrichment analysis by PANTHER showed no enrichments on “Biological Process” and “Molecular Functions” (*P* > 0.05). KEGG pathway analysis showed no significant enrichment in any pathway (P > 0.05), either. These results suggested that these shared germline SNPs were unlikely to be functionally relevant to LC.

### Individual germline SNPs and PPI network patterns showed significant association with familial lung cancer

**Fig. 2:**
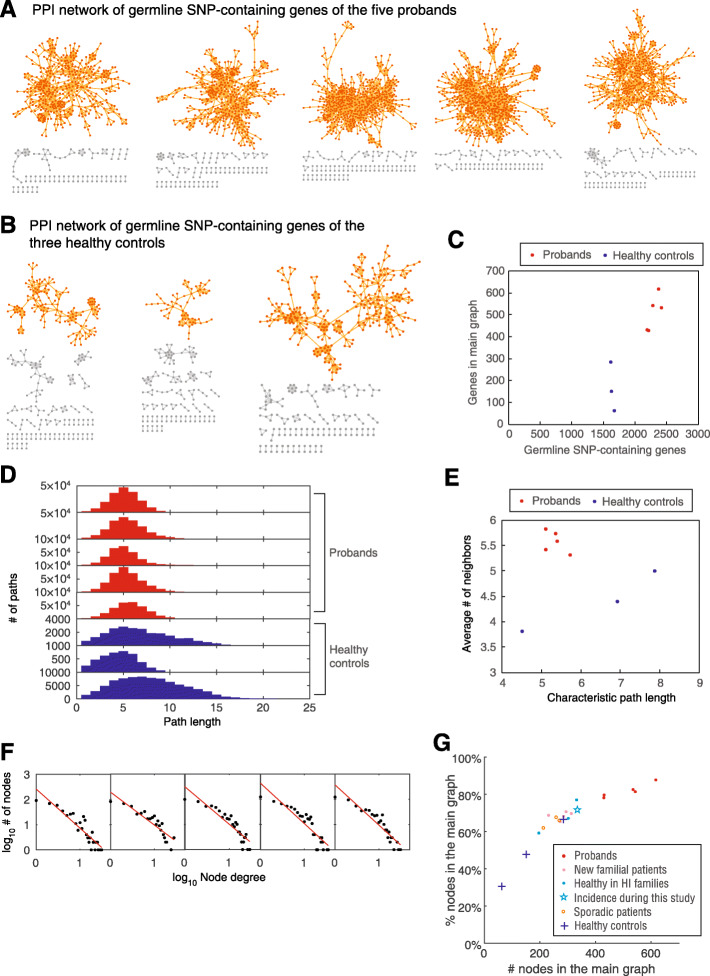
PPI network analysis of genes containing germline SNPs. **a** PPI network of germline SNP-containing genes of the five probands. Each node represents a gene containing a germline SNP, and each edge represents a PPI recorded in STRING-DB. Main graphs (the largest interconnected subgraphs) are colored in orange. **b** PPI network of germline SNP-containing genes of the three healthy controls. Main graphs are colored in orange. **c** Numbers of germline SNP-containing genes and genes in the main graphs. **d** Shortest path distribution in the main graphs. **e** Characteristic path length and average number of neighbors in the main graphs. **f** Node degree distribution of the main graphs of the five probands. Red lines are fits according to the power law. **g** The number of nodes in the main graph versus the percentage of nodes in the main graph over the total nodes. The five probands are colored in red. The five patients from independent high-incidence (HI) families are colored in pink. The three healthy individuals from high-incidence (HI) families are colored in cyan; the one who was diagnosed with lung cancer during this study, is shown with a star. Three patients with sporadic lung cancer are shown in orange circles. Three healthy controls are shown with blue crosses

We next performed PPI analyses for genes containing germline SNPs in each proband and healthy control. Most of the genes containing germline SNPs in each of the five probands formed a large and interconnected PPI network main graph (Fig. 2a), whereas those from healthy controls formed much smaller PPI network graphs (Fig. 2b, c). These results demonstrated that germline SNP-containing genes in the probands tended to interact with each other, expanding the impact of SNPs throughout the system and indicating the robustness of the effect.

In addition to many more nodes in the main graph, the proband main graphs also had a much shorter path length than those of the healthy controls, except for healthy control #2 due to the very small main graph for this individual (Fig. 2d, e). Additionally, the proband main graph possessed a significantly higher number of neighbors than that of the healthy controls (*P* = 0.0145, two-tailed Kolmogorov-Smirnov test, Fig. 2e). These results demonstrated that the information on the proband main graphs could be rapidly transmitted to the entire network. Moreover, the degree distribution of the five probands did not strictly follow the power law, with the number of medium-degree nodes markedly higher than that expected by power law (Fig. 2f), indicating that these main graphs were more densely interconnected than a standard biological PPI network (described as scale-free network that obeys the power law).

### Validation of individual germline SNPs and PPI network patterns in other five familial lung cancer patients

If this hypothesis was true, we could deduce that other members in the familial LC families, especially newly diagnosed patients with LC, should share similar features of germline SNPs due to similar genetic backgrounds. The germline SNPs of other five similar probands, four healthy individuals in the former familial families and three patients with sporadic LC were used as a validation set. Similar to the five probands, the latter five familial lung cancer patients generally had many interconnected SNP-containing genes as a large main graph, and the main graph contained more than 60% of the SNP-containing genes (Fig. 2g). This significantly distinguished these individuals from healthy controls (*P* = 0.0485, two-tailed Mann-Whitney U-test). We also tested three patients with sporadic lung adenocarcinoma. These patients had significantly fewer nodes in the main graph than individuals in the familial LC families (*P* = 0.018, two-tailed Mann-Whitney U-test), but were similar to the healthy individuals in the familial LC families (*P* = 0.70, two-tailed Mann-Whitney U-test).

### SNP-containing genes in PI3K/AKT pathway

The highly interconnected SNP-containing genes in familial LC families suggested that these genes may function together in a more effective way by interfering with entire pathways and thus potentially elevating the risk of cancer incidence. As a verification, the five probands shared only two shared KEGG pathways in the top 10 pathways: “Pathways in Cancer” and the “PI3K/AKT Pathway” (Supplementary Table S6A). Similarly, both pathways appeared in the top 10 pathways in the five newly diagnosed patients with LC from other familial families. In sharp contrast, the PI3K/ATK pathway did not appear in the top 10 pathways in three of four healthy individuals in familial families, potentially explaining why these individuals had not yet been diagnosed with LC at the time of participation in the study. This scenario was similar to that for the three healthy controls with no cancer incidence in their families for three generations; only one person had the abovementioned two pathways enriched in the top 10 KEGG pathways. We also analyzed the germline SNPs of three patients with sporadic LC. Interestingly, “Pathways in Cancer” existed in all three patients, whereas the PI3K/AKT pathway was identified in two patients.

In addition, nonsynonymous somatic mutations in the five familial family probands and the three patients with sporadic LC shared the same trends in enriched pathways; that is, the “Pathways in Cancer” or “PI3K/AKT Pathway” appeared in the top 10 KEGG pathways (Supplementary Table S6B). This indicated that somatic mutations in these pathways further reinforced the alterations in these pathways needed to drive the entire system into a cancerous state.

### Number of SNP-containing genes in the PI3K/AKT pathway

**Fig. 3:**
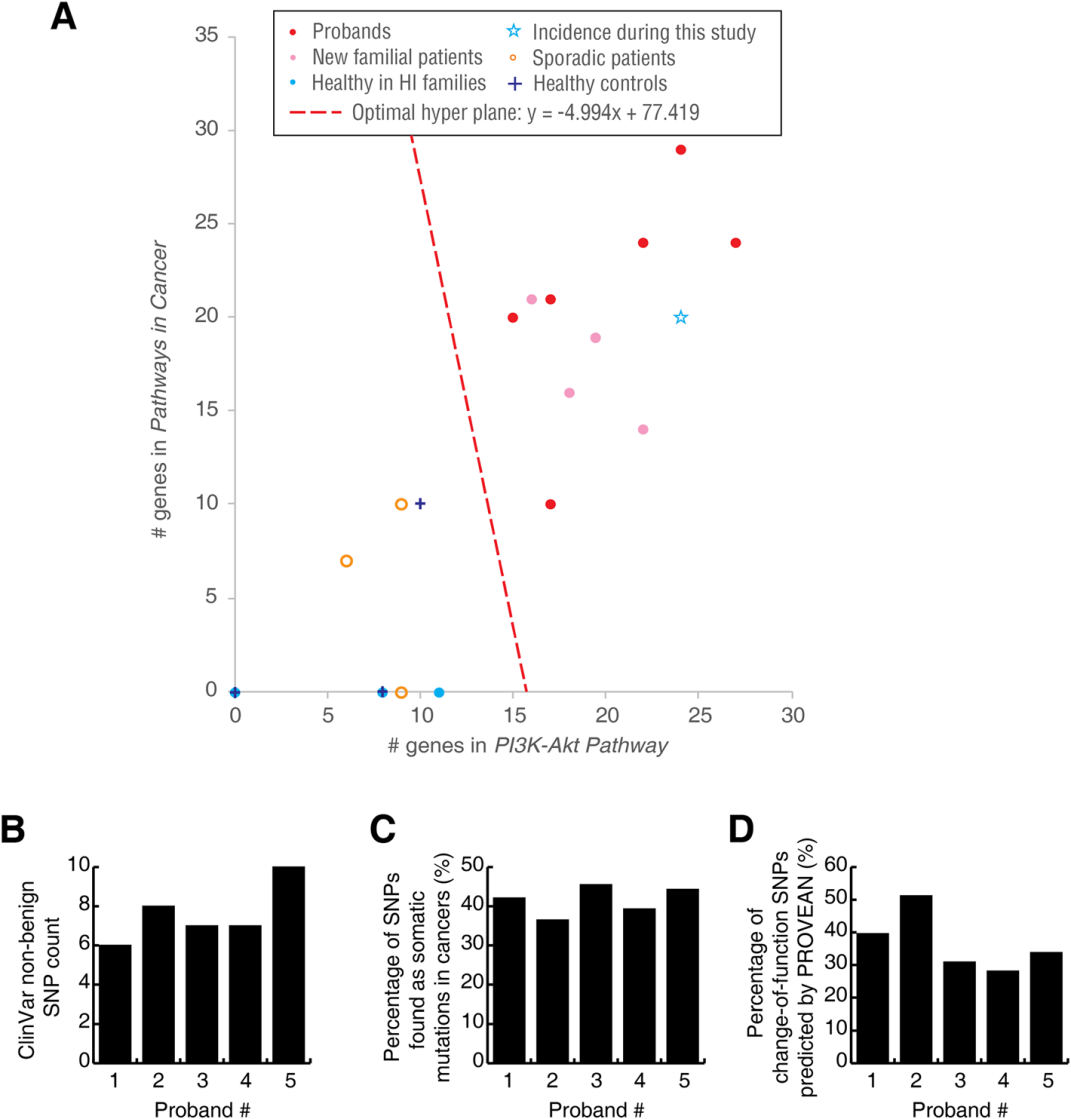
The germline SNP-containing genes in the PI3K/AKT pathway and pathways in cancer. **a** The number of germline SNP-containing genes in the two pathways. The colors and symbols are the same as in Fig. 2g. **b** Number of non-benign PI3K/AKT pathway SNPs recorded in ClinVar database. **c** Percentage of PI3K/AKT pathway SNPs which are found as somatic mutations in cancers (COSMIC database). **d** Percentage of “damaging” (change-of-funciton) PI3K/AKT SNPs, predicted by PROVEAN web server

The numbers of SNP-containing genes in the “Pathways in Cancer” and “PI3K/AKT pathway” were positively correlated (Fig. 3a). The data points were automatically clustered into two groups using the unsupervised hierarchical clustering method way: all five probands and the five newly diagnosed patients with familial cancer had more than 15 SNP-containing genes in the PI3K/AKT pathway and more than 10 genes in “Pathways in Cancer”. In contrast, most healthy individuals (including all three healthy controls and three healthy individuals in familial families) and all three patients with sporadic LC had fewer SNP-containing genes in these two pathways. Thus, the number of germline variation-containing genes of the PI3K/AKT pathway (> 15 genes) may be an important predictor of the high risk of LC. The optimal division line is indicated in Fig. 3 and was solved by a simple SVM classification method.

The functions of these SNPs have not been investigated thoroughly. Nevertheless, we subjected the SNPs of the 5 probands in the PI3K/AKT pathway to functional predictions and database search. In the ClinVar database, 6 ~ 10 SNPs were recorded as non-“benign”, e.g. pathogenic, Conflicting interpretations of pathogenicity, risk factor, etc. (Fig. 3b), which indicated that these SNPs are potential risk factors of diseases (most of which are tumors). In the COSMIC database, nearly half of these SNPs have been found as somatic mutations in cancer (Fig. 3c), indicating that these mutations might be helpful for the cancerous malignancy. We also predicted the functions of these SNPs using SIFT & PROVEAN tool {Choi, 2015 #62}. 28.1 ~ 51.2% of the SNPs were predicted as “damaging” by PROVEAN, which means that these SNPs would alter the protein structures and thus may lead to significant functional changes. These results suggested that the PI3K/AKT SNPs of these familial LC patients may contribute to systemic and functional risk.

### One individual had many germline variations in the PI3K/AKT pathway

Notably, one healthy individuals in a familial family (marked with arrows in Fig. 2g, Fig. 3 and Supplementary Table S6) exhibited features identical to those of patients with familial LC, including a large and interconnected main graph of the germline SNP-containing genes (Fig. 2g), “Pathways in Cancer” and the PI3K/AKT pathway as the top two KEGG pathways (Supplementary Table S6), and 24 and 20 SNP-containing genes in the two pathways, respectively (Fig. 3). One year after her initial enrollment in this study, cancer lesions were detected in her lungs, and pathological adenocarcinoma was diagnosed. Although more cases are needed for reinforcement, this case indicated the feasibility of using such criteria to predict the incidence of familial LC.

## Discussion

Our previous study showed that an increased risk of LC was associated with the number of affected relatives [21]. The risk of LC development is significantly higher in patients with adenocarcinoma with familial aggregation. Further analysis of these results indicated that familial risks are compatible with genetic predisposition but can also reflect shared exposures and genetic factors.

As a highly complex disease, LC cannot be explained by single specific mutations. Highly variable somatic mutations may provide a temporal and limited explanation for the progression, but not the incidence of LC. Our results also showed that no shared somatic mutations were found in the five probands. In contrast, germline SNPs can be indicators of the susceptibility to the disease. The GWAS-identified susceptibility loci of LC only showed their marginal statistical significance to incidence, suggesting that a rational combination of many genetic loci may be suitable for predicting LC incidence, particularly in the context of familial LC.

Based on the concepts of systems biology, we aimed to screen germline SNP networks that may contribute to familial LC. We confirmed that, despite differences among SNPs in the familial LC probands, these patients shared the same enrichment in the PI3K/AKT pathway, highlighting this pathway as a major predictor of familial susceptibility to LC.

The PI3K activates multiple downstream pathways such as RAS, ERK and mTOR pathways, which are crucial for protein synthesis, cell survival, cell growth and proliferation [22–24]. Somatic alterations including mutations and amplification in genes in the PI3K pathway, such as PTEN, PIK3CA, PIK3R1, and AKT, are often found in various kinds of human cancers including lung and activate the PI3K/AKT pathway, driving carcinogenesis [24, 25]. Genetic alterations of PI3K pathway were rarely reported in familial lung cancer [26]. Other some specific loci or genes in the genome, like 6q23–25, ARHGEF5 were also reported in familial lung cancers [27, 28]. The actual function of these genetic or genomic alteration needs further investigation. Many PI3K/AKT pathway inhibitors were designed as therapeutic treatment for multiple cancer categories [23, 29]. In contrast, germline variations in the PI3K pathway, particularly the combinatory effects of multiple SNPs in this pathway, are often overlooked. Notably, individual germline SNPs in the PI3K pathway rather than shared SNPs or somatic mutations were found to be related to familial LC in this study.

Germline SNPs are not the direct cause of LC, and most patients with familial LC did not harbor known driver somatic mutations. Therefore, one possible explanation for the function of these disperse germline SNPs is as follows: germline SNPs in these patients may provide a fragile “network basis” of nonsynonymous SNP-containing genes enriched in the PI3K/AKT pathway. Although single SNP possess minor malignant potency, accumulation of many such SNPs collectively contribute to the susceptibility in a perceptible significance, which has been evidenced in the studies on same-sex sexual behavior and cognition capabilities [13–15]. Networking of such SNP-containing genes may promote the PI3K/AKT pathway to an unstable or precancerous status, resulting in susceptibility to cancer initiation. The fragile network will collapse into imbalance and increase the risk of cancer development if further somatic mutations occur. These somatic mutations are not necessarily the driver mutations, but together with the fragile germline-determined PI3K/AKT pathway, this nonrobust system will easily become unbalanced with random environmental fluctuations and may develop in an emergent and/or chaotic manner, resulting in cancer. This hypothetical explanation of the basic role of PI3K/AKT SNPs in cancer is echoed by a series of system biology approaches. For example, alterations in CDK1 and CDK2 enzyme kinetics parameters will disrupt the regular cell cycle [29]; the in vitro tumor cell proliferation dynamics follows a fractal structure different from normal oscillatory dynamics [30]. Although mathematical nonlinear theories are thought to model carcinogenesis in pure theoretical approaches [31], our results may provide explicit and experimental support of this philosophy. This theory may also apply to other types of cancer.

Compared with the well-known Knudson “two-hit hypothesis”, which emphasizes the subsequent deactivation of the two alleles of tumor-suppressor genes, our “mutation network basis hypothesis” emphasized the interactions of the SNP-involved gene sets, not a single tumor-suppressor gene. Compared with Nordling’s “multimutation theory”, which assumed that the genesis of cancer requires the accumulation of multiple consecutive mutations, our “mutation network basis hypothesis” emphasized the importance of the inherited fragile network due to germline SNPs. Therefore, the “two-hit hypothesis” and “multimutation theory” may be more suitable to explain the incidence of sporadic cancer.

Although our study was limited by the small number of familial LC pedigrees due to the rare occurrence of pedigrees with such strict criteria, our results provided insights into the management of familial susceptibility to LC based on several concepts. First, accurate whole-exome or whole-genome sequencing, not just genetic testing of a small gene panel or several specific SNPs, should be applied to everyone during early life to evaluate risk at a systems level. The decreasing price of sequencing makes this approach affordable. Second, in cases of a high risk of familial incidence, the individual should adjust his/her lifestyle to avoid inducing factors, such as smoking, air pollution and mutagens. Third, high-risk populations should undergo more frequent screening to detect early-stage tumors. Finally, healthy individuals in families with familial LC should undergo such WES tests because they may share the same fragile germline basis as the probands. This echoes a recent study that the population genomic screening of all young adults is extremely cost-effective in disease prevention and enhancing life quality [32]. Our results suggested that such WES-level genomic screening might be more useful in the familial LC families.

## Conclusions

In summary, the phenomenon that the enrichments of germline SNPs in the PI3K/AKT pathway might be a major predictor of familial susceptibility to LC.

## Supplementary Information

**Additional file 1.** Methods. Support vector machine (SVM) classification.

**Additional file 2: Fig. S1.** Protein-protein interaction (PPI) network constructed using the shared germline mutated genes of the five LC probands. Disconnected genes were removed from the graph.

**Additional file 3: Fig. S2.** Bioinformatic workflow of next-generation sequencing data processing. FET = Fisher exact test.

**Additional file 4: Table S1.** The questionnaire of the living environment.

**Additional file 5: Table S2.** The demographic and histologic of probands.

**Additional file 6: Table S3.** Summary of sequencing reads and mapping of the reads.

**Additional file 7: Table S4.** Somatic mutations of probands.

**Additional file 8: Table S5.** Shared germline mutations of probands.

**Additional file 9: Table S6.** Top 10 KEGG pathways of the non-synonymous mutated genes of the individuals.

## Data Availability

The datasets generated during and analyzed during the current study are not publicly available due to privacy or ethical restrictions but are available from the corresponding author on reasonable request. FANSe2: http://chi-biotech.com/fanse2/

## Abbreviations

LC: Lung cancer
ORs: Odds ratios
CIs: Confidence intervals
SNV: Single-nucleotide variation
KEGG: Kyoto Encyclopedia of Genes and Genomes
SNPs: Single-nucleotide polymorphisms
PPI: Protein-protein interaction
SVM: Support vector machine
