# Supplementary material for: Germline variation networks in the PI3K/AKT pathway corresponding to familial high-incidence lung cancer pedigrees
Source: BMC Cancer. 2020 Dec 9;20:1209. doi: 10.1186/s12885-020-07528-3 (PMC7724858; doi:10.1186/s12885-020-07528-3)
Supplement: Supplementary file 1 — Additional file 1. Methods. Support vector machine (SVM) classification. [file 12885_2020_7528_MOESM1_ESM.docx]

**Supplementary Methods**

**Support vector machine (SVM) classification**

SVM open source LIBSVM (R package “e1071”, www.r-project.org) was applied to draw decision boundaries between data points from healthy individuals and pedigrees with familial high risk of LC.18 Specifically, SVMs based on the Kernel and parameter C regression technique were trained by a set of data with the “leave-one-out” cross-validation approach. Each assessment used one sample from the original sample group as the validation sample, with the remaining samples serving as the training data; this procedure was repeated to ensure that each sample was used only once as a validation sample in the SVM training step. Then, the trained model was used to determine the decision boundaries between healthy individuals and pedigrees with familial high risk of LC. After training steps, the linear Kernel with the best regularization parameter C was chosen as our feature set due to the data point pattern. Based on the combination of optional γ and C values, the influence without the training set was sufficient for a decision hyperplane.
