# Supplementary figures and images for: Germline variation networks in the PI3K/AKT pathway corresponding to familial high-incidence lung cancer pedigrees
Source: BMC Cancer. 2020 Dec 9;20:1209. doi: 10.1186/s12885-020-07528-3 (PMC7724858; doi:10.1186/s12885-020-07528-3)

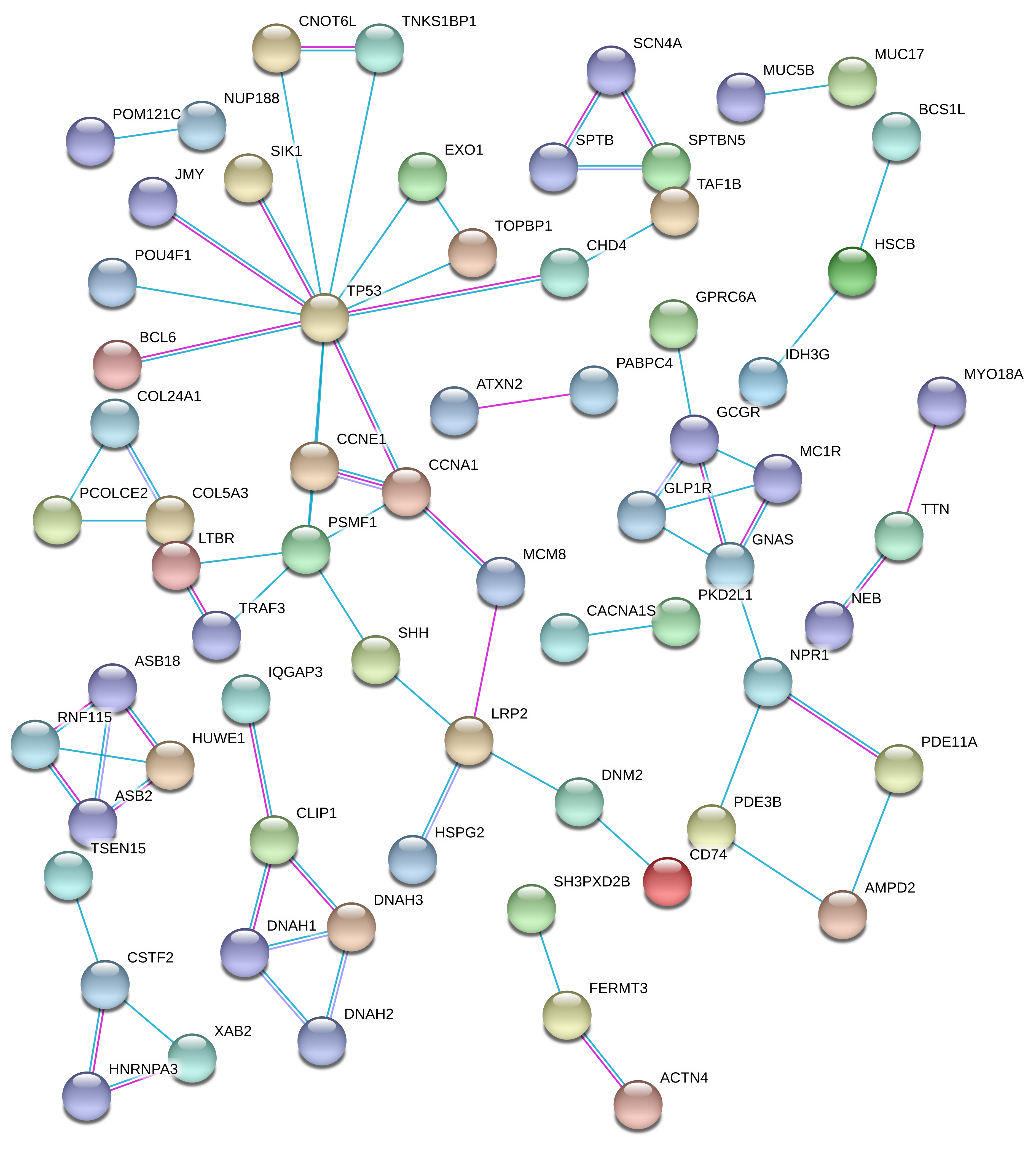

Supplement: Supplementary file 2 — Additional file 2: Fig. S1. Protein-protein interaction (PPI) network constructed using the shared germline mutated genes of the five LC probands. Disconnected genes were removed from the graph. [file 12885_2020_7528_MOESM2_ESM.png]

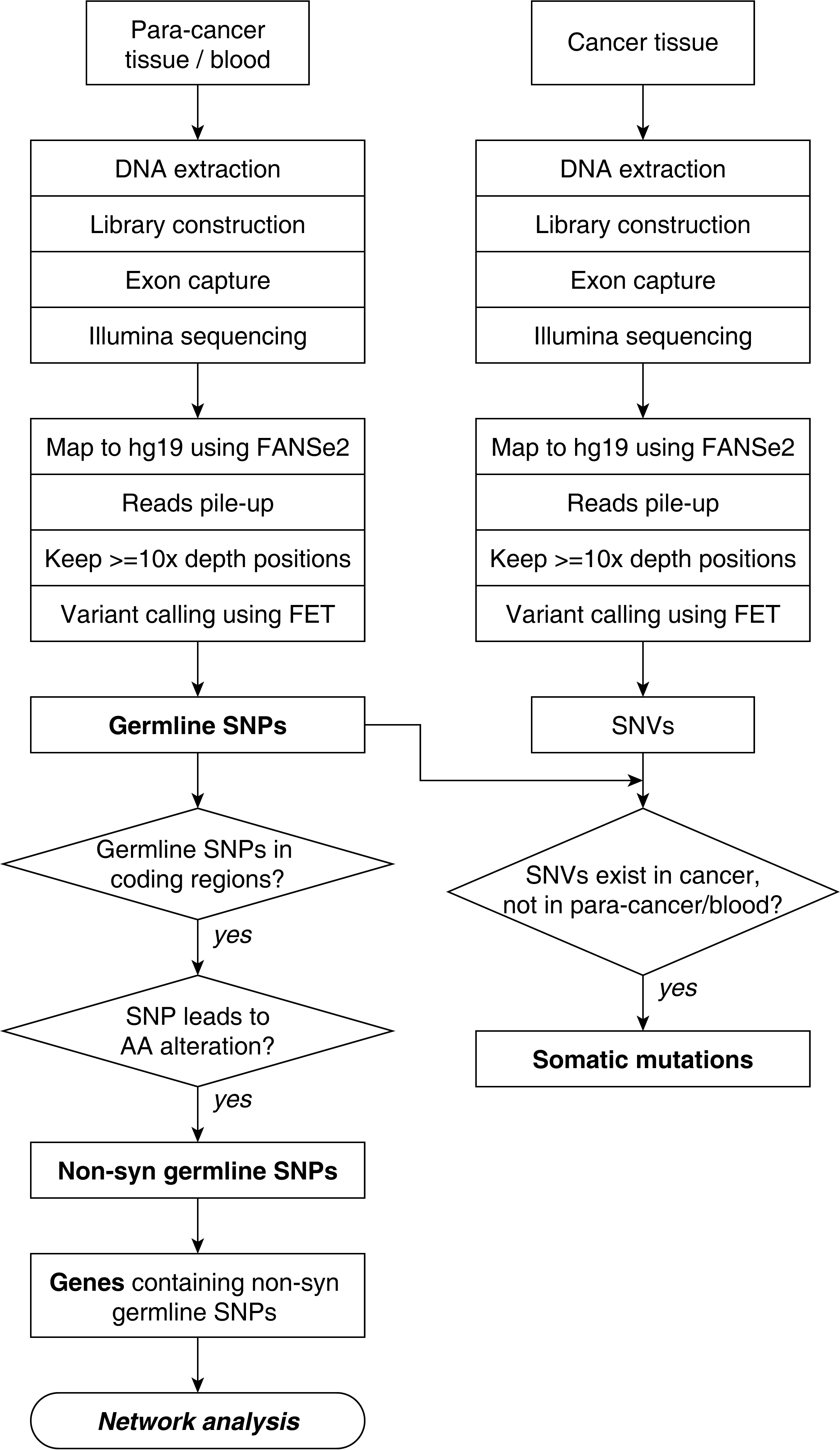

Supplement: Supplementary file 3 — Additional file 3: Fig. S2. Bioinformatic workflow of next-generation sequencing data processing. FET = Fisher exact test. [file 12885_2020_7528_MOESM3_ESM.tif]
