# Supplementary material for: Germline variation networks in the PI3K/AKT pathway corresponding to familial high-incidence lung cancer pedigrees
Source: BMC Cancer. 2020 Dec 9;20:1209. doi: 10.1186/s12885-020-07528-3 (PMC7724858; doi:10.1186/s12885-020-07528-3)
Supplement: Supplementary file 4 — Additional file 4: Table S1. The questionnaire of the living environment. [file 12885_2020_7528_MOESM4_ESM.docx]

**Table S1 The questionnaire of the living environment**

|  | Level | Duration | Score |
| --- | --- | --- | --- |
| Cooking practices | Olive and peanut |  | 0 |
|  | Soybean and rapeseed | <10 years  10-20 years  >20years | 0  1  2 |
| Fuel use | Gas and coal | <5 years  5-10 years  >10years | 0  1  2 |
|  | Other |  | 0 |
| Housing characteristics(kitchen ventilation) | Good/fairly good |  | 0 |
|  | Poor | <10 years  10-20 years  >20years | 0  1  2 |
| Tobacco smoke exposure(Household residents smoking or  Co-worker smoking habits at work) | Never |  | 0 |
|  | Ever | <5 years  5-10 years  >10years | 0  1  2 |
| Whether they lived in a polluted industrial zone. | Never |  | 0 |
|  | Ever | <5 years  5-10 years  >10years | 0  1  2 |

Light : 0-3; middle: 4-7; : heavy: 8-10.

We collected information on age, gender, race, lung disease history, living environment, occupational exposure, and smoking history for probands and controls using a structured questionnaire. The living environment was supplemented by detailed questions on cooking practices, fuel use, housing characteristics, tobacco smoke exposure, and whether or not the subject lived in a polluted industrial zone. The selection of intensity was classified as never, light, and heavy based on the questionnaire (Supplementary Table S1). Lung diseases were defined as asthma, silicosis, lung abscesses, bronchiectasis, tuberculosis, pulmonary fibrosis, emphysema, and chronic bronchitis. The questions pertaining to occupational exposure included whether or not the subject usually had contact with dust, asbestos, radioactive materials, and volatile chemicals for each work period of at least 1 year. All jobs with no potential exposure to lung cancer carcinogens were excluded. Then, the name of the company, type of company, period, and information about what was being produced in the department was used, as the probability of exposure to some carcinogens differed between companies and between periods. Each job title, specified by period and company, was scored separately according to the probability of exposure to four carcinogens (asbestos, paint dust, PAHs, and welding fumes, especially stainless-steel welding). Three exposure categories were defined, as follows: no exposure to the specific carcinogen; possible exposure (probability of exposure estimated to be <80%);and nearly certain exposure(probability of exposure >80%). We determined the living environment or occupational exposure as positive if any of the selections were considered heavy.
