# Supplementary material for: Germline variation networks in the PI3K/AKT pathway corresponding to familial high-incidence lung cancer pedigrees
Source: BMC Cancer. 2020 Dec 9;20:1209. doi: 10.1186/s12885-020-07528-3 (PMC7724858; doi:10.1186/s12885-020-07528-3)
Supplement: Supplementary file 5 — Additional file 5: Table S2. The demographic and histologic of probands. [file 12885_2020_7528_MOESM5_ESM.docx]

**Supplementary Table S2 Demographic and Histologic Characteristics of Probands**

| Characteristic | Probands 01 | Probands 02 | Probands 03 | Probands 04 | Probands 05 |
| --- | --- | --- | --- | --- | --- |
| Age, years | 76 | 61 | 59 | 44 | 61 |
| Race | Han | Han | Han | Han | Han |
| Marital Status | Married | Married | Married | Married | Married |
| Smoker | Never | Light | Heavy | Never | Heavy |
| Lung Disease history | Never | Never | Never | Never | Never |
| Living Environment | Never | Never | Ever | Never | Never |
| Occupational exposure | Never | Never | Never | Ever | Never |
| Pathlogy | Adenocarcinoma | Adenocarcinoma | Adenocarcinoma | Adenocarcinoma | Adenocarcinoma |
