# Supplementary material for: Germline variation networks in the PI3K/AKT pathway corresponding to familial high-incidence lung cancer pedigrees
Source: BMC Cancer. 2020 Dec 9;20:1209. doi: 10.1186/s12885-020-07528-3 (PMC7724858; doi:10.1186/s12885-020-07528-3)
Supplement: Supplementary file 6 — Additional file 6: Table S3. Summary of sequencing reads and mapping of the reads. [file 12885_2020_7528_MOESM6_ESM.docx]

**Supplementary Table S3: Summary of sequencing reads and mapping of the reads. A paired-end read is considered as two single end reads.**

|  | Sample | Sequencer | read length | total reads | mapped reads | mapped% | nominal avg depth (x) |
| --- | --- | --- | --- | --- | --- | --- | --- |
| Familial lung cancer probands | #1 cancer | HiSeq 2000 | 100 | 103561176 | 87582901 | 84.57% | 91.23 |
|  | #1 para-cancer | HiSeq 2000 | 100 | 135037354 | 114206461 | 84.57% | 118.97 |
|  | #2 cancer | HiSeq 2000 | 100 | 130384758 | 110601545 | 84.83% | 115.21 |
|  | #2 para-cancer | HiSeq 2000 | 100 | 122820852 | 104318897 | 84.94% | 108.67 |
|  | #3 cancer | HiSeq 2000 | 100 | 175856872 | 148984203 | 84.72% | 155.19 |
|  | #3 para-cancer | HiSeq 2000 | 100 | 199122968 | 163760116 | 82.24% | 170.58 |
|  | #4 cancer | HiSeq 2000 | 100 | 195176766 | 161494869 | 82.74% | 168.22 |
|  | #4 para-cancer | HiSeq 2000 | 100 | 172014290 | 142149807 | 82.64% | 148.07 |
|  | #5 cancer | HiSeq 2000 | 100 | 111864572 | 92230227 | 82.45% | 96.07 |
|  | #5 para-cancer | HiSeq 2000 | 100 | 127925408 | 106079156 | 82.92% | 110.50 |
| Healthy persons in high-incidence lung cancer families | #1 | HiSeq 2000 | 100 | 121074162 | 96173081 | 79.43% | 100.18 |
|  | #2 | HiSeq 2000 | 100 | 130321816 | 101659491 | 78.01% | 105.90 |
|  | #3 | HiSeq 2000 | 100 | 124490606 | 102437772 | 82.29% | 106.71 |
| Healthy controls with no cancer incidence for 3 generations | #1 | HiSeq 2500 | 125 | 77216848 | 68412401 | 88.60% | 89.08 |
|  | #2 | HiSeq 2500 | 125 | 77833172 | 68781010 | 88.37% | 89.56 |
|  | #3 | HiSeq 2500 | 125 | 79165284 | 67657898 | 85.46% | 88.10 |
| Sporadic lung cancer patients | #1 cancer | HiSeq 2500 | 125 | 83280132 | 69796558 | 83.81% | 90.88 |
|  | #1 para-cancer | HiSeq 2500 | 125 | 82163392 | 68230713 | 83.04% | 88.84 |
|  | #2 cancer | HiSeq 2500 | 125 | 83240496 | 68151882 | 81.87% | 88.74 |
|  | #2 para-cancer | HiSeq 2500 | 125 | 80136410 | 67203932 | 83.86% | 87.51 |
|  | #3 cancer | HiSeq 2500 | 125 | 82685856 | 69233253 | 83.73% | 90.15 |
|  | #3 para-cancer | HiSeq 2500 | 125 | 82216216 | 67388470 | 81.96% | 87.75 |
| Newly emerged familial patients | #1 | HiSeq 2500 | 125 | 79724448 | 68317398 | 85.69% | 88.95 |
|  | #2 | HiSeq 2500 | 125 | 76692932 | 58824319 | 76.70% | 76.59 |
|  | #3 | HiSeq 2500 | 125 | 83651306 | 67314605 | 80.47% | 87.65 |
|  | #4 | HiSeq 2500 | 125 | 81584674 | 66027594 | 80.93% | 85.97 |
|  | #5 | HiSeq 2500 | 125 | 81592466 | 68019225 | 83.36% | 88.57 |
| New incidence during follow-up |  | HiSeq 2500 | 125 | 83133568 | 67197412 | 80.83% | 87.50 |
